# Demographic Amplification of Climate Change Experienced by the Contiguous United States Population during the 20^th^ Century

**DOI:** 10.1371/journal.pone.0045683

**Published:** 2012-10-24

**Authors:** Jason Samson, Dominique Berteaux, Brian J. McGill, Murray M. Humphries

**Affiliations:** 1 Department of Natural Resource Sciences, Macdonald Campus, McGill University, Ste-Anne-de-Bellevue, Québec, Canada; 2 Biology Department, Université du Québec à Rimouski, Rimouski, Québec, Canada; 3 School of Biology and Ecology Sustainability Solutions Initiative, University of Maine, Orono, Maine, United States of America; University of Copenhagen, Denmark

## Abstract

Better understanding of the changing relationship between human populations and climate is a global research priority. The 20^th^ century in the contiguous United States offers a particularly well-documented example of human demographic expansion during a period of radical socioeconomic and environmental change. One would expect that as human society has been transformed by technology, we would become increasingly decoupled from climate and more dependent on social infrastructure. Here we use spatially-explicit models to evaluate climatic, socio-economic and biophysical correlates of demographic change in the contiguous United States between 1900 and 2000. Climate-correlated variation in population growth has caused the U.S. population to shift its realized climate niche from cool, seasonal climates to warm, aseasonal climates. As a result, the average annual temperature experienced by U.S. citizens between 1920 and 2000 has increased by more than 1.5°C and the temperature seasonality has decreased by 1.1°C during a century when climate change accounted for only a 0.24°C increase in average annual temperature and a 0.15°C decrease in temperature seasonality. Thus, despite advancing technology, climate-correlated demographics continue to be a major feature of contemporary U.S. society. Unfortunately, these demographic patterns are contributing to a substantial warming of the climate niche during a period of rapid environmental warming, making an already bad situation worse.

## Introduction

The changing relationship between human populations and climate is of major interest given persistent population growth, accelerating climate change, and increasingly complex and diversified influences of climate on human well-being. While historical climate change is known to have had profound impacts on human populations [1], [2], [3], [4], [5], the impact of contemporary climate change on our societies is likely to be more complex and regionalized because of the diversity of technological, economic and social conditions influencing the human-climate relationship [6], [7], [8]. The complexity of human societies and the rapidity of their demographic and technological transitions make it likely that relationships between human populations and climate have and will continue to change over time. In particular, various forms of technological, economic and social development could mean that the density and population growth of contemporary human populations is less related to climate and more related to socioeconomic variables than was historically the case. For example, access to climate controlled buildings combined with technological advances in food production, transportation, and storage might allow humans to spread into diversified climatic niches that were previously unsuitable for food production and thermal comfort, thereby weakening the correlation between demographics and climate. Alternatively, if technological, economic and social development contributes to concentrated population growth in particular regions and climate zones, then correlations between climate and population growth may persist or even strengthen over time.

The contiguous United States during the last century represents an ideal place and time for evaluating the changing relationship between humans and climate. During the 20^th^ century, the total population size of the United States increased, in a highly spatially heterogeneous fashion, from 76 million in 1900 to 281 million in 2000 [9]. The availability of detailed census data collected every decade with a high degree of spatial resolution provides rich and robust data on demographic trends. Historical climate conditions can be inferred with reasonable confidence and spatial resolution given adequate temporal and spatial coverage of the instrumental weather record. Finally, the availability of additional socio-economic variables, obtained directly or derived from census data, allows examination of non-climate correlates of demographic patterns.

Here we quantify the evolving climate niche of the contiguous United States population during a century of accelerating demographic, socio-economic, and climate change by combining interpolated climate data with county-based demographic and socio-economic trends during five time periods in the 20^th^ century. We quantify how a century of demographic change has altered the relationship between human population density and climate. In particular, we assess whether the mean climate exposure of the contemporary U.S. population has become cooler or warmer, wetter or drier during the last century. We do so by combining county-based population estimates and county-interpolated climate data to estimate the mean climate experienced by the U.S. population at five points in time during the 20^th^ century. This analysis generates a climate niche surface reflecting the number of people experiencing a given combination of climate conditions, which is prone to change over time as the climate changes and the number of people living in different climatic regions changes. We conclude the paper by comparing the influence of climate and non-climate correlates of U.S. population growth over the course of the 20^th^ century. The spatially heterogeneous nature of demographic change, and its potential climate and non-climate correlates, requires a statistical framework capable of modelling regional differences in estimated relationships. Systems with such regional disparities have been defined as non-stationary [10]. Here we use geographically weighted regression (GWR), a non-stationary technique [11], to examine spatial relationships between county-based demographic change and four climate variables and four non-climate variables over the 20^th^ century, to assess whether the importance of climate-correlated demographics has increased or decreased over time.

## Materials and Methods

### Human population data

We estimated the population density for each county in the contiguous U.S. by dividing its total population size by its area based on U.S. censuses [9]. Although the first U.S. census was done in 1790, we contrasted demographic patterns on a 20-year basis during the 20^th^ century because comprehensive climate data were not available prior to 1900. Between 1900 and 2000, the number of U.S. counties increased from 3063 to 3141 and the geographical boundaries of some counties shifted. Given the difficulty in comparing population figures between censuses when county boundaries are shifting [12], we restricted our analyses to the 2728 counties that kept the same geographical boundaries and that had census data available throughout the last century. Such partial sampling of U.S. censuses during the 20^th^ century has been shown to adequately represent the demographic patterns of the whole country [13]. Given our interest in the demographic response to spatially and temporally variable climatic and non-climatic conditions, we used human density annual growth rate instead of absolute change in population size our analysis. We calculated human density annual growth rate, hereafter referred to as demographic growth rate, for each county during each 20-year interval with the following equation:
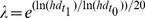
Where *λ* represents demographic growth rate, *hd* represents human density and *t_0_* and *t_1_* represent the first and last year of the interval (e.g. 1900 and 1920), respectively. Note that from equation 1, population growth rate is mathematically independent of population density, and is therefore free to vary negatively or positively with (or be unrelated to) population density [14].

### Climate

We used 1901–2000 gridded monthly time series of temperature and precipitation data (available at http://climate.geog.udel.edu/~climate/html_pages/archive.html) to calculate four climatic variables representing the average and seasonality of climate conditions across the United States in the last century (average annual temperature (°C), total annual precipitation (mm), standard deviation of monthly average temperature (°C), and standard deviation of monthly total precipitation (mm)). For each climatic variable, hereafter referred to as annual temperature, annual precipitation, temperature seasonality, or precipitation seasonality, we averaged yearly estimates over the 20 years of each temporal horizon. We then interpolated these climates variables using an inverse distance weighting technique and extracted climate conditions at the centroid of each county.

### Quantifying temporal changes in climate exposure

We first estimated the average climate conditions experienced by U.S. citizens across all counties by weighting the climate conditions of each county by its population size. We then used local regressions (LOESS) to represent local population density in climate space and to identify peaks of density throughout the century. We smoothed the data using 2-dimensional LOESS regression with second order polynomials. A smoothing parameter of alpha = 0.3 was used but the results were qualitatively similar using different smoothing parameters.

### Assessing climatic and non-climatic correlates of population growth

To assess changes in the relative importance of climate and non-climate correlated population growth over the 20^th^ century, we combined the four climate variables, describing the average and seasonality of both temperature and precipitation, with four potential non-climate correlates of population growth into a single model. The non-climate variables we consider are limited to those that could be estimated on a county-by-county basis for each 20-year interval between 1900 and 2000.

Income and population density are two important socioeconomic correlates of demographic patterns [15], which were available from U.S. population census data dating back to 1900, and thus were included in the analysis. We used human density in each county at the beginning of each temporal horizon to represent the influence of initial population density on demographic growth rates. As many counties had low densities and few had very high densities throughout the century, we log_10_ transformed the variable *human density* to normalize its distribution.

A comprehensive and unbiased measure of income was difficult to obtain because the economic queries in the U.S. censuses of the twentieth century were not consistent. The most comprehensive data available in each census were wages in the manufacturing sector for 1900 and 1940, wages of wage earners for 1920, and categorical personal income for 1980. No comprehensive economic data was available for 1960. We standardized income estimates by calculating county's z-scores within census to preserve the geographical differences in income while allowing a direct comparison between censuses. We interpolated z-scores from 1940 and 1970 censuses to obtain income estimates for 1960 with a weighted average where 1940 income estimates had a weight of 0.333 and 1970 income estimates had a weight of 0.667. A similar interpolation was done for 26% of the counties for the year 1940 because they did not have any income estimate. The 1980 census reported income as the number of persons represented by income range (e.g. 5000–7500$, 7500–10 000$, etc.) so we calculated average income for each county as the sum of the product of the number of persons in each category and the median income of that category. We refer to the county's z-score of income estimate during the first year of each temporal horizon as the variable *income* in all our analyses.

Given the likely importance of agriculture and food production to human population distribution [16], [17] and the availability of historical crop and pasture extent from the ISLSCP II Historical Land Cover and Land Use (1700–1990) [18], we included *agricultural density* (calculated as arc-sine square root transformed crop and pasture extent divided by county land area) at the beginning of each temporal horizon as an additional non-climate variable.

Finally, because human population size and distribution have been previously argued to be strongly associated with coastal zones and navigable rivers and only weakly with climate conditions [19] we included *distance from waterway* (calculated as the shortest distance between the centroid of each county and the Atlantic Ocean, Pacific Ocean, or the Great Lakes and St-Lawrence River system; square root transformed to normalize its distribution) as the fourth non-climate variable.

A frequent challenge in assessing non-climatic correlates of population growth or distribution is the availability of variables quantified across the same spatial and temporal resolutions as climate variables [20], [21]. Frequently, many variables are known to contribute to population-level outcomes, but the lack of temporally and spatially-explicit quantification of these variables makes it difficult to demonstrate their importance. A complete analysis of socio-economic correlates of human population growth would include a much broader suite of variables than we examine here, including additional indicators of wealth, education, employment, and health status. However, as far as we are aware, these indicators are not available country-wide, at a county-level of resolution, dating back to 1900 and available through to 2000. This is a serious limitation, because excluding important non-climatic predictors of population growth may artificially elevate the predictive power of climate variables, particularly if non-climatic drivers are themselves correlated with climate variables. Thus, our analysis of non-climate correlates of human population growth must be viewed as incomplete, and interpreted not as a complete examination of climatic and non-climatic contributors to population growth at any one point in time, but rather as an assessment of changes over time in the relative importance of a select few potential climatic and non-climatic correlates of population growth. Of course many more climatic factors could also have been included but are not. In the end, it is well known that including more variables in one category of variables than another can bias results to suggest the category with more variables is more important [22]. From this perspective having four climate variables and four socio-economic variables is optimal and does not bias in either direction.

We used geographically weighted regression (GWR) to describe the spatial non-stationarity of the relationships between demographic growth rates and climatic and non-climatic variables. Although similar to standard regression models, GWR allows spatial flexibility in regression coefficients by providing a unique regression model for each location based on a geographical weighting function. Take, for example, a model predicting demographic growth rates (λ) based on four variables (V_1_, V_2_, V_3_, V_4_). Demographic growth rates are then predicted by the following spatially-explicit regression model:
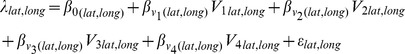
Local regression coefficients are estimated as:
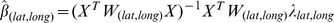
where *X* represents the matrix of predictors and *W* represents the matrix of geographical weights for each of the observed data used at a given location. We used a bi-square geographical weighting function as shown in equation 4:
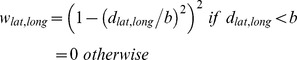
where *w* represents the weight of observed datum, *d* is the distance between the observed datum and the area where local regression parameters are estimated, and *b* is a threshold distance referred to as the bandwidth. The bandwidth is limited to a minimum value by high spatial colinearity in predictor values while very large bandwidths cannot describe non-stationary patterns. As an absolute bandwidth can create biases in coastal areas given the smaller sample size used to estimate their regression coefficients, we used an adaptive kernel bandwidth where the weights are geographically adjusted to represent 30% of the neighbouring counties. This adaptive bandwidth represented, on average, 9.7° of latitude and longitude. The high spatial colinearity between some variables required us to increase the bandwidth of three models (GWR_NC_ for 1901–1920 and 1921–1940: 50% bandwidth, GWR_NC_ for 1941–1960: 45% bandwidth). See Table S1 for a comparison between stationary and non-stationary models. All GWR analyses were done with the software SAM [23].

## Results

**Figure 1:**
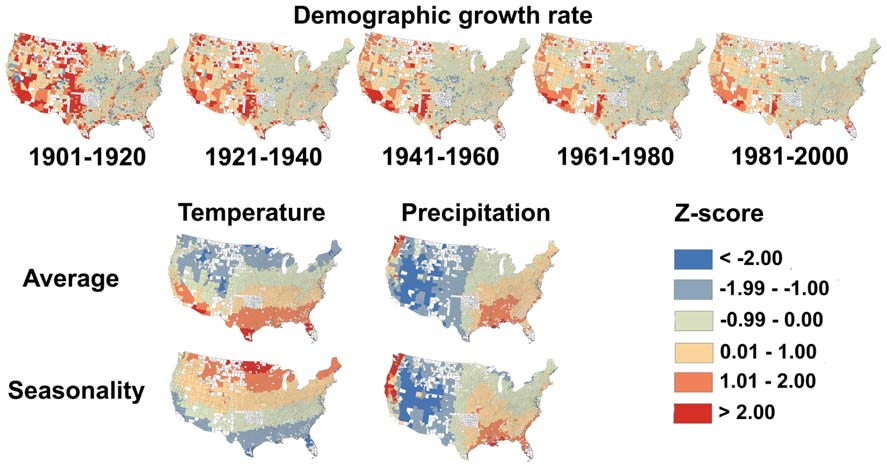
Spatial patterns of demographic growth rate and climate conditions during the 20^th^ century. Counties shown in white were not included in the analyses because they did not have consistent census data or changed their geographical boundaries in the 20^th^ century. Temporal changes are shown based on five 20-year intervals for demographic growth rate whereas climatic variables are only shown for the 1981–2000 interval because these variables remained very similar throughout the 20^th^ century (see fig. S1 for the non-climate variables and Fig. S6 for temporal changes in the spatial patterns of climate conditions). In order to directly compare the spatial patterns between variables, each panel represents county z-scores based on the average and standard deviation of that variable throughout the century. A z-score of 0 represent the mean, whereas a value of 1 represent one standard deviation above the mean.

**Figure 2:**
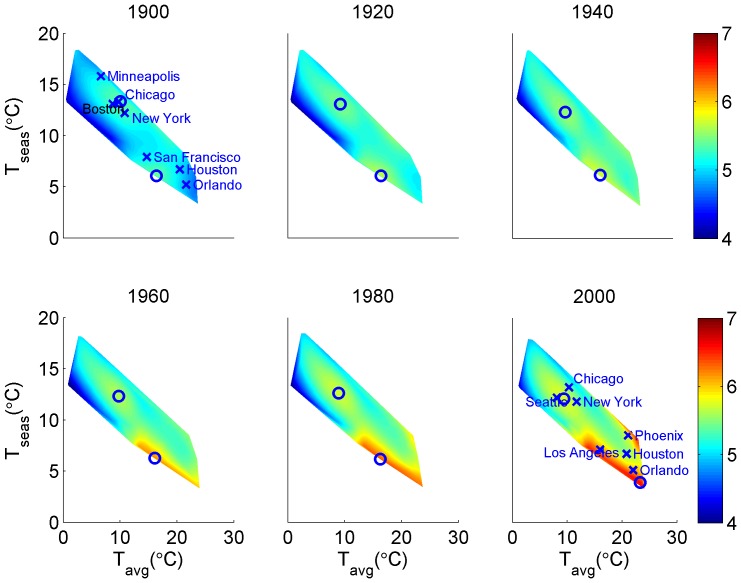
Variation in human abundance across the thermal niche of U.S. populations throughout the 20^th^ century. The climate niche is based on average annual temperature (°C) and temperature seasonality (°C). Human abundance data are from the population census of the year displayed on each panel and the colour ramp is log_10_ scaled. The peaks identified with LOESS are shown as circles. We estimated the climate conditions of each temporal horizon by averaging annual climate conditions of the preceding 20 years and, given the lack of climate data prior to 1900, we used the 1901–1920 climate averages in our analyses of both 1900 and 1920. See Fig. S4 for a similar analysis based on precipitation.

**Figure 3:**
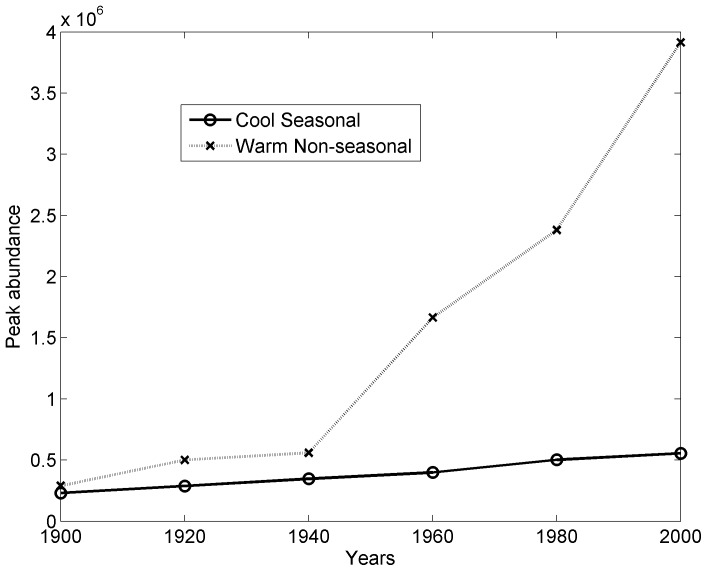
Population abundance of the two population peaks of the U.S. thermal niche during the 20^th^ century.

**Figure 4:**
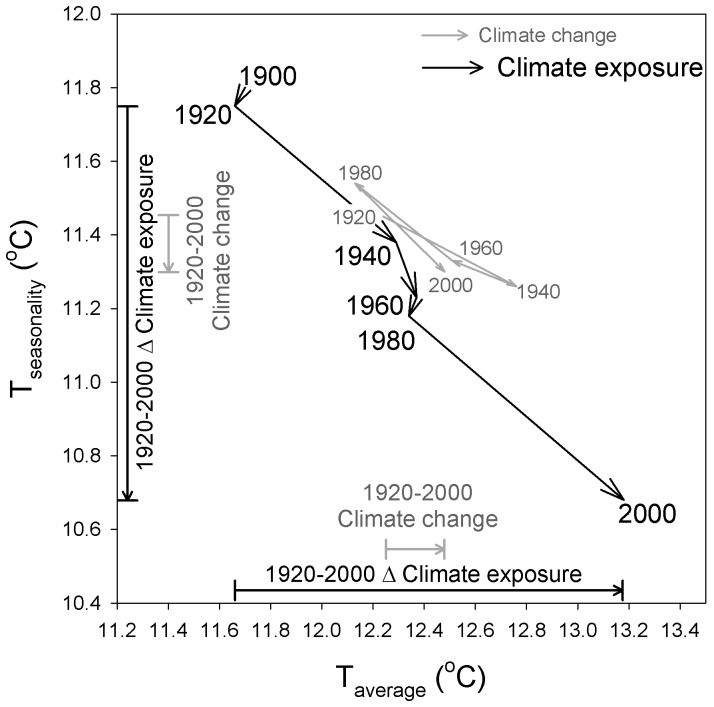
Changes in U.S. climate conditions averaged across 2728 counties (*Climate change*) and averaged across U.S. citizens (*Climate exposure*). The x-axis represents average annual temperature (°C) and the y-axis temperature seasonality (°C). The arrows beside the axes represent the change in climate conditions between 1920 and 2000 for both time series. We estimated the climate conditions of each temporal horizon by averaging annual climate conditions of the preceding 20 years and, given the lack of climate data prior to 1900, we used the 1901–1920 climate averages in our analyses of both 1900 and 1920. The *Climate change* result for 1900 is therefore omitted while the *Climate exposure* results in 1900 and 1920 are based on the same climate conditions but different population sizes. See Fig. S5 for a similar analysis based on precipitation.

The climate niche of the contiguous U.S. population has changed dramatically during the 20^th^ century as a result of climate-correlated and regionalized demographic trends (Fig. 1). Plotting population abundance in climate space, defined by average annual temperature and temperature seasonality, reveals a two-peak climate niche throughout the 20th century, with a cool, seasonal peak corresponding to climate conditions typical of the Middle Atlantic region and a warm, aseasonal peak corresponding to a southern belt extending from Florida to California (Fig. 2). Throughout most of the 20^th^ century, both peaks were relatively stationary in climate space and, concomitant with nationwide population growth, increased in abundance. However, the warm, aseasonal population peak increased in abundance much more than the cold, seasonal peak (Fig. 3), particularly between 1980 and 2000 when its location also shifted to the extreme warm and aseasonal edge of U.S. climate space. As a result, the average temperature experienced by U.S. citizens has increased by more than 1.5°C between 1920 and 2000, when climate change accounted for only a 0.24°C increase (Fig. 4). Meanwhile, the temperature seasonality experienced by U.S. citizens decreased by 1.1°C between 1920 and 2000 during a time period when temperature seasonality decreased by 0.15°C.

**Figure 5:**
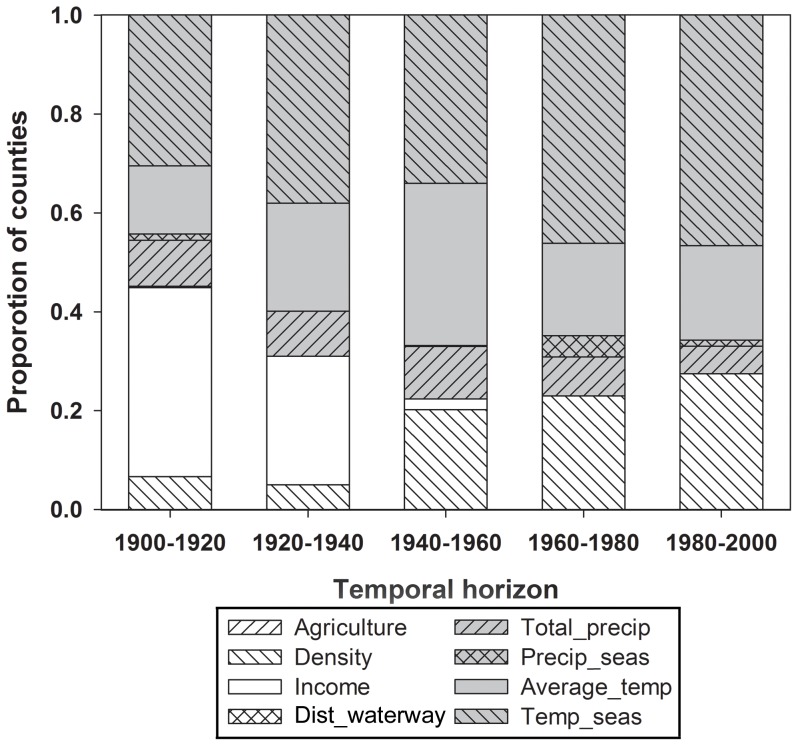
Relative importance of climatic and non climatic variables in GWR models predicting demographic growth rates. Climatic variables are shown in grey and non-climatic variables in white. The relative importance of each variable is based on the proportion of counties where its standardized regression coefficient (stdβ) was highest in absolute value (see Fig. 6).

**Figure 6:**
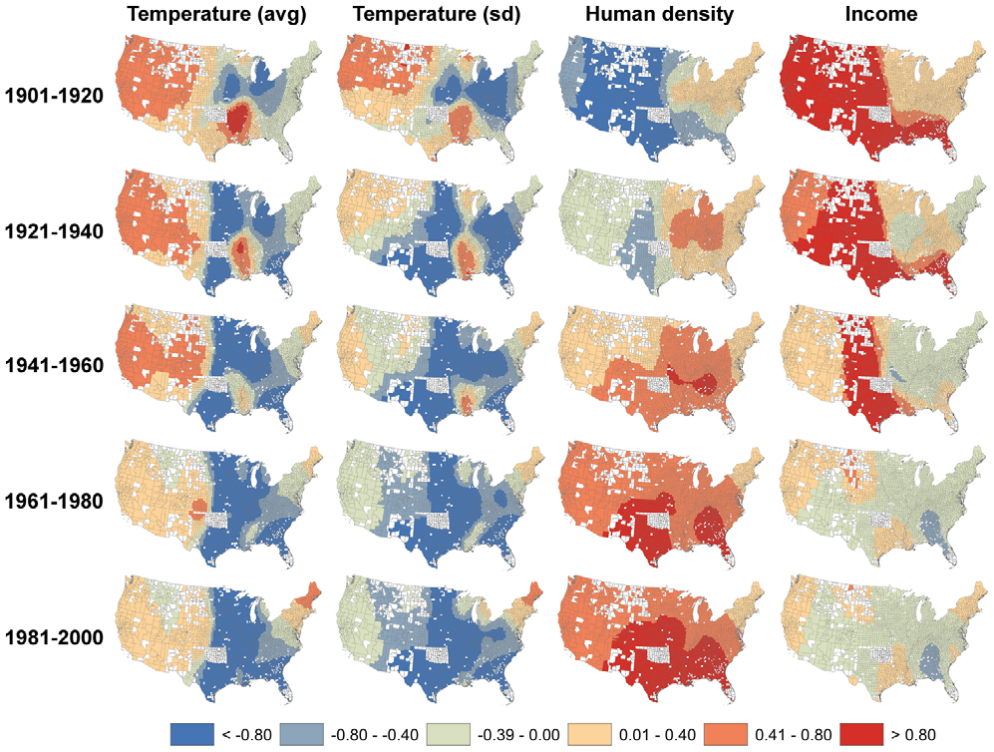
Standardized regression coefficients (Stdβ) of the four most important predictors of demographic growth rates. Counties shown in white were not included in the analyses because they did not have consistent census data or changed their geographical boundaries during the 20^th^ century. See Fig. S7 for the Stdβ of the four other variables used in this analysis.

Comparison of four climatic and four non-climatic correlates of population growth provides strong evidence of persistent climate-correlated demographic trends in the U.S. throughout the 20^th^ century. The relative importance of climate variables as predictors of population growth rate strongly increased from 1900 to 1960, then remained important from 1961 to 2000 (Fig. 5). Early in the century, population growth was most pronounced in the western half of the U.S. (Fig. 1) and positively correlated with warm regions of low human density and high income (Fig. 6). A positive, but weakening association between population growth and annual temperature persisted in warmer portions of the west for the remainder the 20^th^ century. However, the negative relationship between population density and growth prevailing in the west early in the century shifted in the latter half of the century to a strongly positive association between density and growth, spanning the entire U.S. but particularly strong in the south. Thus, areas that were already densely populated grew more than areas that were less densely populated, which tended to maintain and amplify the initial importance of climate as a correlate of population growth.

## Discussion

A strong knowledge of historical patterns is essential to comprehend the current state of a system and to anticipate how this system may change in the future [24]. The socio-economic and environmental conditions of most human societies have drastically changed during the 20^th^ century [21] and the contiguous United States has been particularly transformed both by rapid demographic growth and socio-economic development [25], [26], [27]. These changes have been highly spatially heterogeneous across the country and, more importantly, the relationships between changes in demographic growth, socio-economic factors, and climate conditions have also been highly spatially heterogeneous.

Our analysis has shown that human density increased preferentially in the warmest and least thermally seasonal regions of the United States and that the pace of this thermal niche displacement accelerated throughout the 20^th^ century. These climate-correlated demographics have strongly shifted the thermal niche of human populations in the contiguous United States, greatly increasing the warm climate exposure experienced by American citizens. While average annual temperature has increased during the 20^th^ century by 0.65°C across the globe [7], and by 0.24°C across the contiguous United States, spatially heterogeneous demographic growth has caused the climate experienced by U.S. residents to increase by 1.5°C. Given these warm regions are also generally dry, climate-correlated demographics have caused the U.S. population to shift its realized climate niche towards drier conditions, experiencing 46 mm less annual precipitation over a period when annual precipitation increased by 27 mm (Fig. S4 and S5). Pronounced and sustained climate anomalies are apparent in Fig. 4 and Fig. S5, including the 1932–1939 dustbowl era that manifests as a pronounced warming and drying of the climate between 1920 and 1940 [28] and the 1940–1969 cooling phase that manifests as a gradual cooling of the climate between 1940 and 1980 [29]. However, these figures also clearly show how climate-correlated demographics can serve to either counter or amplify climate change. For example, temperature exposure remained essentially constant from 1940–1980 despite sustained climate cooling, because of disproportionate population growth in warm regions. But when the climate switched to a warming phase in 1970 and yet warm climate population growth persisted, we entered an era of demographic amplification of climate change. Between 1980 and 2000, the average annual temperature exposure of the U.S. population has increased 2.6 times more than the thermometer has warmed. These results provide a robust historical framework to better evaluate the potential consequences of anticipated climate change, demographic growth and water stress on human well-being in the United States [30], [31], [32], [33].

Comparison of climatic and non-climatic correlates of U.S. demographic patterns indicates the importance and persistence of climate-correlated population growth throughout the 20^th^ century, that is particularly important during the last half of the century. This trend is supported by the proportion of counties in which a climate variable was the strongest correlate of growth, which ranged from slightly more than half in 1900–1920 to more than two thirds from 1940 onwards (Fig. 5). Further, climatic correlates have remained in the same order of importance and directionality over the last century, whereas the most important non-climatic correlates have changed in importance and sign (Fig. 6). For example, income was an important positive correlate of growth in the west during the first half of the century. Since then, and coincident with the great U-turn in income inequality in the United States [34], income-correlated growth has declined (and even became negative in the Southeast), while the importance of human density has grown nationwide with the highest density counties being now characterized by the highest per capita population growth, especially in southern regions. This post-1950's trend towards urbanization and agglomeration is well described and has been in part attributed to technological developments that reduced “the constraints of geographic space and the costs of concentration” [35]. Overall, then, populations have grown most rapidly in the warmest, least seasonal and most densely populated regions of the United States. During a century of radical technological and societal change, climate-correlated population growth has been a persistent feature in U.S. demographic patterns.

Readers may reasonably question whether we assume causation underlies the correlations between climate and demography that we identify here. Do we mean to suggest that climate is a direct determinant of population growth; such that, like potted plants, human populations grow in response to temperature and water? Or do we mean to suggest that population growth occurs in particular times and places for reasons that have nothing to do with prevailing environmental conditions, such that climate correlations persist only as artefacts or coincidence? The first and most rigorous answer is that we do not know, because we have not conducted the research necessary to resolve why the U.S. population has grown when and where it did and we are not aware of a body of research that compares the relative influence of environmental and societal contributors to population growth in historical and contemporary societies (but see [36]). The second and less rigorous answer is that, in the absence of direct evidence, we speculate these correlations reflect neither direct causality nor complete coincidence. Our speculation is pushed to the broad middle ground between these endpoint extremes by, on one hand, the many social, economic and historical factors known to shape where humans live, how well they survive, and how much they reproduce [25], [26], [27]. On the other hand, recognition of the fundamental influence of climate on our thermal comfort, food supply, lifestyle, infrastructure, and environmental hazards [37] makes us hesitant to dismiss climate as a merely coincidental condition in human affairs. The third and most pertinent answer is that resolving the basis of these correlations is, for present purposes, less important than documenting their strength and persistence. Regardless of why population growth is correlated with climate in the contiguous U.S., the strength and persistence of this correlation throughout the last century, in a region and a time with great potential for departure from the climate constraints and dependencies that have affected human populations in the past [2], [3], [4], [38], [39], [40], suggests climate-correlated demography will continue to be an important contributor to climate exposure in the future.

Our analysis provides a rare “hindcast” in which historical change in regional density can be quantified with robust data [3], [4]. Our analyses of five 20-year intervals show important and regionally coherent changes in demographic growth and its climatic and non-climatic correlates throughout the century. These results reinforce the importance of forecasted demographic changes in our assessements and mitigation of human vulnerability to climate change [41], including how population redistribution to warmer, drier regions will exacerbate recent and precicted increases in water stress [30], [31] and electrical demands for thermal comfort through air conditioning [42]. Given that the change in climate exposure observed in this study is in the same direction as the anticipated climate change caused by greenhouse gas emissions [7], it is likely that the economic burden of climate change during this century will be much greater and regionally disparate than predicted because of demographic amplification of climate change. The annual cost of an increase of 1.5°C in average temperature has been estimated at 1.44 billion dollars in a 1990 economy and 4.39 billion dollars in a 2060 economy [43]. By distributing the expected cost across five thermal zones of the contiguous United States, it is estimated that more than 80% of the cost originates from the two warmest zones [43], consistent with cooling being more expensive than heating. More importantly, these predictions of future costs and their regional origins, are based on the assumption of geographically homogenous population increase across the United States between 1990 and 2060 [43], which is unlikely given the 20^th^ century demographic patterns quantified here. Furthermore, the recent emergence of population density as a positive correlate of population growth means that the urban and suburban heat island effect [44] will be an increasingly important contributor to climate exposure in the coming decades, which will further amplify the impacts of disproportionate population growth in warm regions. Climate change predictions should thus explicitly incorporate regional and localized demographic disparities [41] to adequately anticipate the potential impacts of climate change on human well-being. Further, mitigation strategies might reasonably focus on both atmospheric and demographic contributions to experienced climate change.

## Supporting Information

Figure S1 **Spatial patterns of demographic growth rate, climatic variables, biophysical and socio-economic variables during the 20^th^ century.** Counties shown in white were not included in the analyses because they did not have consistent census data or changed their geographical boundaries in the 20^th^ century. Temporal changes are shown based on five 20-year intervals for the first four variables whereas climatic variables and distance from the sea are only shown for the 1981–2000 interval because these variables remained very similar throughout the 20^th^ century (see fig. S6 for temporal changes in the spatial patterns of climate conditions). In order to directly compare the spatial patterns between variables, each panel represents county z-scores based on the average and standard deviation of that variable throughout the century. A z-score of 0 represent the mean, whereas a value of 1 represent one standard deviation above the mean. The *income* z-scores are represented with a different scale based on quantiles to highlight geographical disparities because their distributions were skewed by a few counties with very high income z-scores (see material and methods for details).
(DOCX)

Figure S2 **Standardized regression coefficients of socio-economical variables for 2728 U.S. counties during the 20^th^ century estimated with GWR_NC_ models predicting demographic growth rates.** White counties were not included in the analyses because they did not have consistent census data or changed their geographical boundaries in the 20^th^ century.
(DOCX)

Figure S3 **Standardized regression coefficients of climatic variables for 2728 U.S. counties during the 20^th^ century estimated with GWR_C_ models predicting demographic growth rates.** White counties were not included in the analyses because they did not have consistent census data or changed their geographical boundaries in the 20^th^ century.
(DOCX)

Figure S4 **Variation in human abundance across the precipitation niche of U.S. populations based on 2728 U.S. counties throughout the 20^th^ century.** The climate niche is based on total annual precipitation (mm) and precipitation seasonality (mm). Human abundance data are from the population census of the year displayed on each panel. We estimated the climate conditions of each temporal horizon by averaging annual climate conditions of the preceding 20 years and, given the lack of climate data prior to 1900, we used the 1901–1920 climate averages in our analyses of both 1900 and 1920.
(DOCX)

Figure S5 **Changes in climate conditions in the contiguous United States during the 20^th^ century averaged across 2728 counties (** ***Climate change*** **) and averaged across U.S. citizens (** ***Climate exposure*** **).** The x-axis represents total annual precipitation (mm) and the y-axis precipitation seasonality (mm). The arrows beside the axes represent the change in climate conditions between 1920 and 2000 for both time series. We estimated the climate conditions of each temporal horizon by averaging annual climate conditions of the preceding 20 years and, given the lack of climate data prior to 1900, we used the 1901–1920 climate averages in our analyses of both 1900 and 1920. The *Climate change* result for 1900 is therefore omitted while the *Climate exposure* results in 1900 and 1920 are based on the same climate conditions but different population sizes. See material and methods for details.
(DOCX)

Figure S6 **Spatial patterns of four climate variables for 2728 U.S. counties in five 20-year intervals during the 20^th^ century.** Counties shown in white were not included in the analyses because they did not have consistent census data or changed their geographical boundaries in the 20^th^ century. In order to directly compare the spatial patterns between variables, each panel represents county z-scores based on the average and standard deviation of that variable throughout the century. A z-score of 0 represent the mean, whereas a value of 1 represent one standard deviation above the mean.
(DOCX)

Figure S7 **Standardized regression coefficients (Stdβ) of the four least important predictors of demographic growth rates for 2728 U.S. counties in five 20-year intervals during the 20^th^ century (see** **Fig. 6** **).** Counties shown in white were not included in the analyses because they did not have consistent census data or changed their geographical boundaries during the 20^th^ century. See fig. 6 for the Stdβ of the four other variables used in this analysis.
(DOCX)

Table S1 **Model selection with Akaike information criterion (AIC) of stationary (OLS) and non-stationary (GWR) regression models predicting demographic growth rates for 2728 U.S. counties in the 20^th^ century based on climatic (C) and/or non-climatic (NC) predictors.** The AICc weigths of the GWR_C+NC_ models were always one across all temporal horizons. The non-stationary nature of GWR was taken into account by adjusting the number of effective parameters (ranges of effective parameters across temporal horizons: GWR_C+NC_ = 63.3–65.2; GWR_C_ = 33.1–33.8; GWR_NC_ = 22.9–39.8).
(DOCX)
