# Supplementary material for: Demographic Amplification of Climate Change Experienced by the Contiguous United States Population during the 20th Century
Source: PLoS One. 2012 Oct 24;7(10):e45683. doi: 10.1371/journal.pone.0045683 (PMC3480346; doi:10.1371/journal.pone.0045683)
Supplement: Figure S1 — Spatial patterns of demographic growth rate, climatic variables, biophysical and socio-economic variables during the 20th century. Counties shown in white were not included in the analyses because they did not have consistent census data or changed their geographical boundaries in the 20th century. Temporal changes are shown based on five 20-year intervals for the first four variables whereas climatic variables and distance from the sea are only shown for the 1981–2000 interval because these variables remained very similar throughout the 20th century (see fig. S6 for temporal changes in the spatial patterns of climate conditions). In order to directly compare the spatial patterns between variables, each panel represents county z-scores based on the average and standard deviation of that variable throughout the century. A z-score of 0 represent the mean, whereas a value of 1 represent one standard deviation above the mean. The income z-scores are represented with a different scale based on quantiles to highlight geographical disparities because their distributions were skewed by a few counties with very high income z-scores (see material and methods for details). (DOCX) [file pone.0045683.s001.docx]

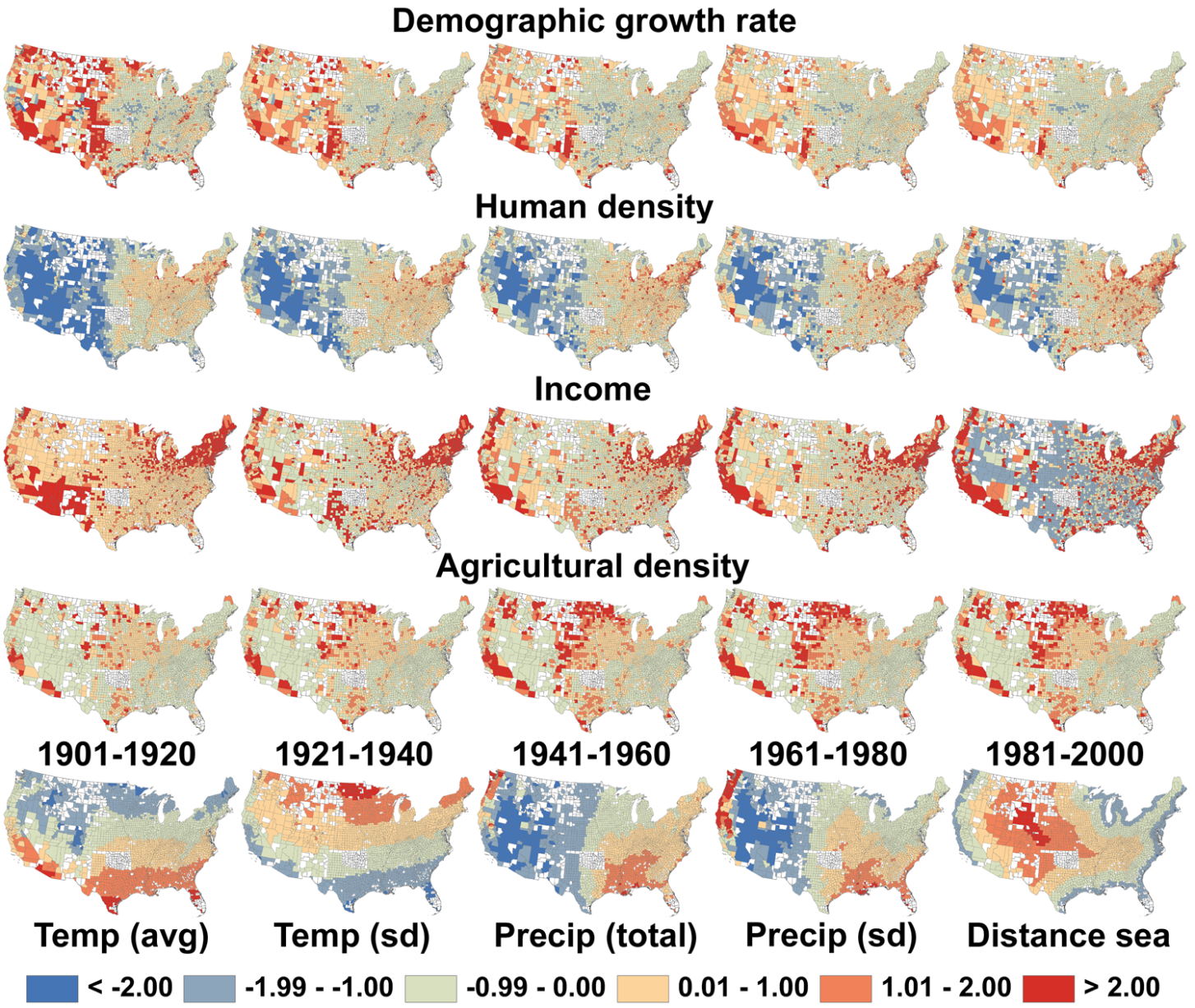


Figure S1. **Spatial patterns of demographic growth rate, climatic variables, biophysical and socio-economic variables during the 20^th^ century**. Counties shown in white were not included in the analyses because they did not have consistent census data or changed their geographical boundaries in the 20^th^ century. Temporal changes are shown based on five 20-year intervals for the first four variables whereas climatic variables and distance from the sea are only shown for the 1981-2000 interval because these variables remained very similar throughout the 20^th^ century (see fig. S6 for temporal changes in the spatial patterns of climate conditions). In order to directly compare the spatial patterns between variables, each panel represents county z-scores based on the average and standard deviation of that variable throughout the century. A z-score of 0 represent the mean, whereas a value of 1 represent one standard deviation above the mean. The *income* z-scores are represented with a different scale based on quantiles to highlight geographical disparities because their distributions were skewed by a few counties with very high income z-scores (see material and methods for details).
