# Supplementary material for: Demographic Amplification of Climate Change Experienced by the Contiguous United States Population during the 20th Century
Source: PLoS One. 2012 Oct 24;7(10):e45683. doi: 10.1371/journal.pone.0045683 (PMC3480346; doi:10.1371/journal.pone.0045683)
Supplement: Figure S2 — Standardized regression coefficients of socio-economical variables for 2728 U.S. counties during the 20th century estimated with GWRNC models predicting demographic growth rates. White counties were not included in the analyses because they did not have consistent census data or changed their geographical boundaries in the 20th century. (DOCX) [file pone.0045683.s002.docx]

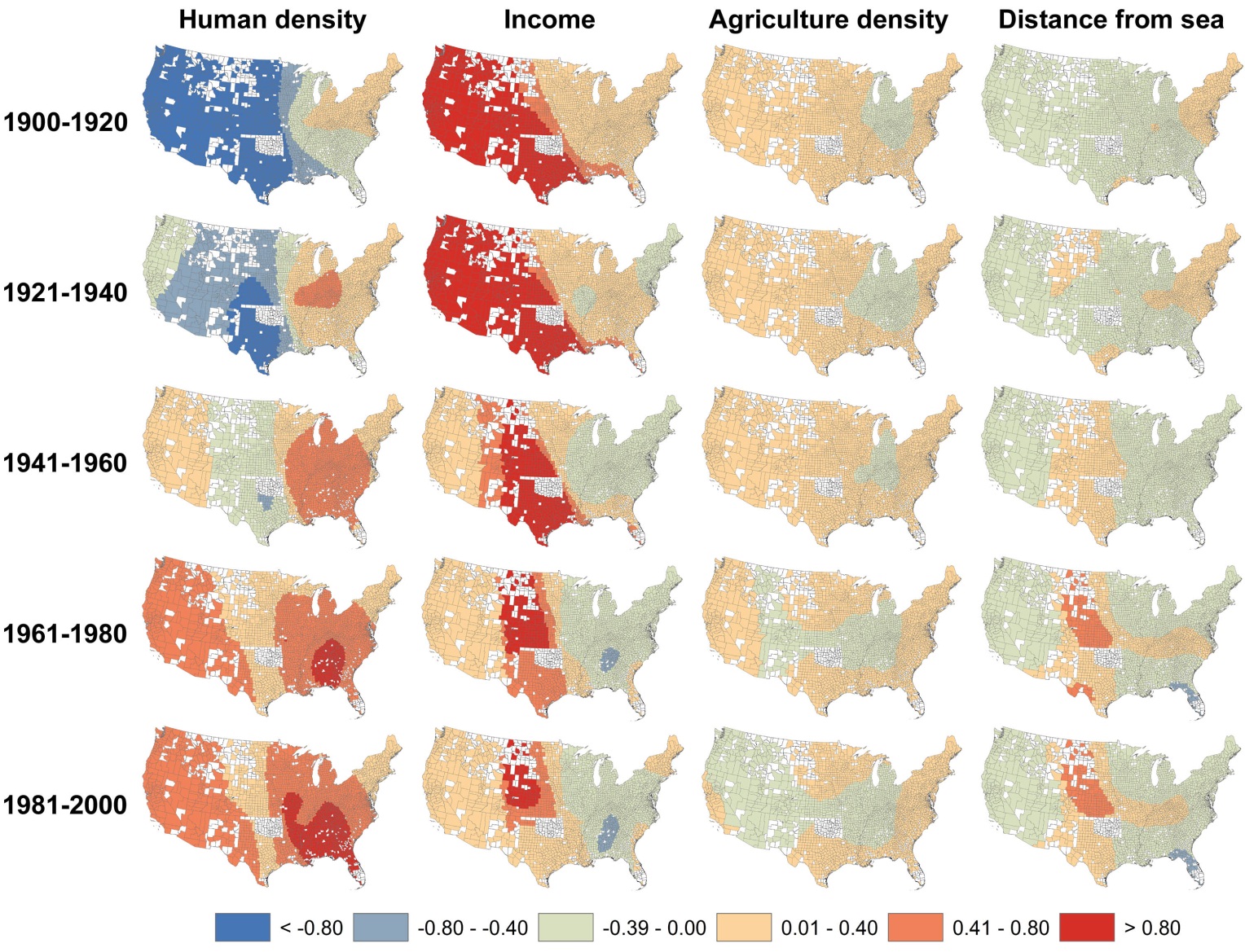


Figure S2. **Standardized regression coefficients of socio-economical variables for 2728 U.S. counties during the 20^th^ century estimated with GWR_NC_ models predicting demographic growth rates.** White counties were not included in the analyses because they did not have consistent census data or changed their geographical boundaries in the 20^th^ century.
