# Supplementary material for: Demographic Amplification of Climate Change Experienced by the Contiguous United States Population during the 20th Century
Source: PLoS One. 2012 Oct 24;7(10):e45683. doi: 10.1371/journal.pone.0045683 (PMC3480346; doi:10.1371/journal.pone.0045683)
Supplement: Figure S4 — Variation in human abundance across the precipitation niche of U.S. populations based on 2728 U.S. counties throughout the 20th century. The climate niche is based on total annual precipitation (mm) and precipitation seasonality (mm). Human abundance data are from the population census of the year displayed on each panel. We estimated the climate conditions of each temporal horizon by averaging annual climate conditions of the preceding 20 years and, given the lack of climate data prior to 1900, we used the 1901–1920 climate averages in our analyses of both 1900 and 1920. (DOCX) [file pone.0045683.s004.docx]

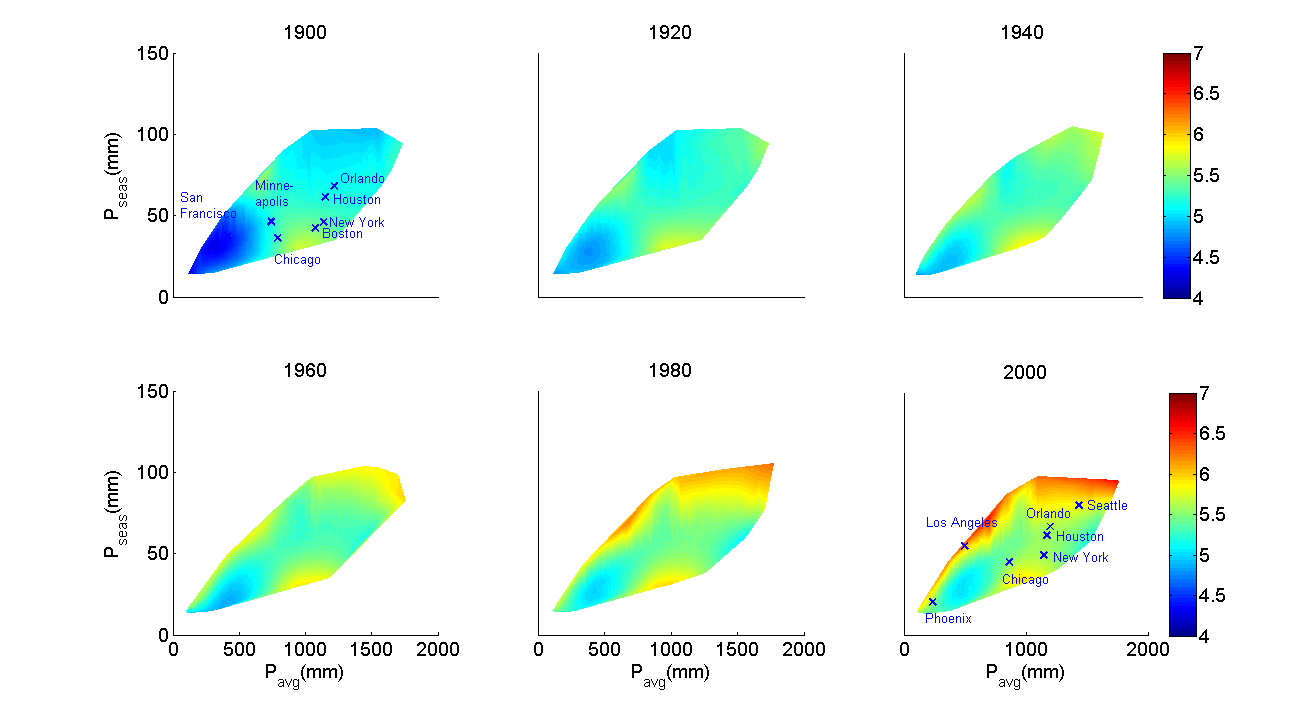


Figure S4. **Variation in human abundance across the precipitation niche of U.S. populations based on 2728 U.S. counties throughout the 20^th^ century.** The climate niche is based on total annual precipitation (mm) and precipitation seasonality (mm). Human abundance data are from the population census of the year displayed on each panel. We estimated the climate conditions of each temporal horizon by averaging annual climate conditions of the preceding 20 years and, given the lack of climate data prior to 1900, we used the 1901-1920 climate averages in our analyses of both 1900 and 1920. The few points P_sd_ >100 mm were removed as they were too sparse in climate space to produce a meaningful interpolation.
