# Supplementary material for: Demographic Amplification of Climate Change Experienced by the Contiguous United States Population during the 20th Century
Source: PLoS One. 2012 Oct 24;7(10):e45683. doi: 10.1371/journal.pone.0045683 (PMC3480346; doi:10.1371/journal.pone.0045683)
Supplement: Figure S5 — Changes in climate conditions in the contiguous United States during the 20th century averaged across 2728 counties ( Climate change ) and averaged across U.S. citizens ( Climate exposure ). The x-axis represents total annual precipitation (mm) and the y-axis precipitation seasonality (mm). The arrows beside the axes represent the change in climate conditions between 1920 and 2000 for both time series. We estimated the climate conditions of each temporal horizon by averaging annual climate conditions of the preceding 20 years and, given the lack of climate data prior to 1900, we used the 1901–1920 climate averages in our analyses of both 1900 and 1920. The Climate change result for 1900 is therefore omitted while the Climate exposure results in 1900 and 1920 are based on the same climate conditions but different population sizes. See material and methods for details. (DOCX) [file pone.0045683.s005.docx]

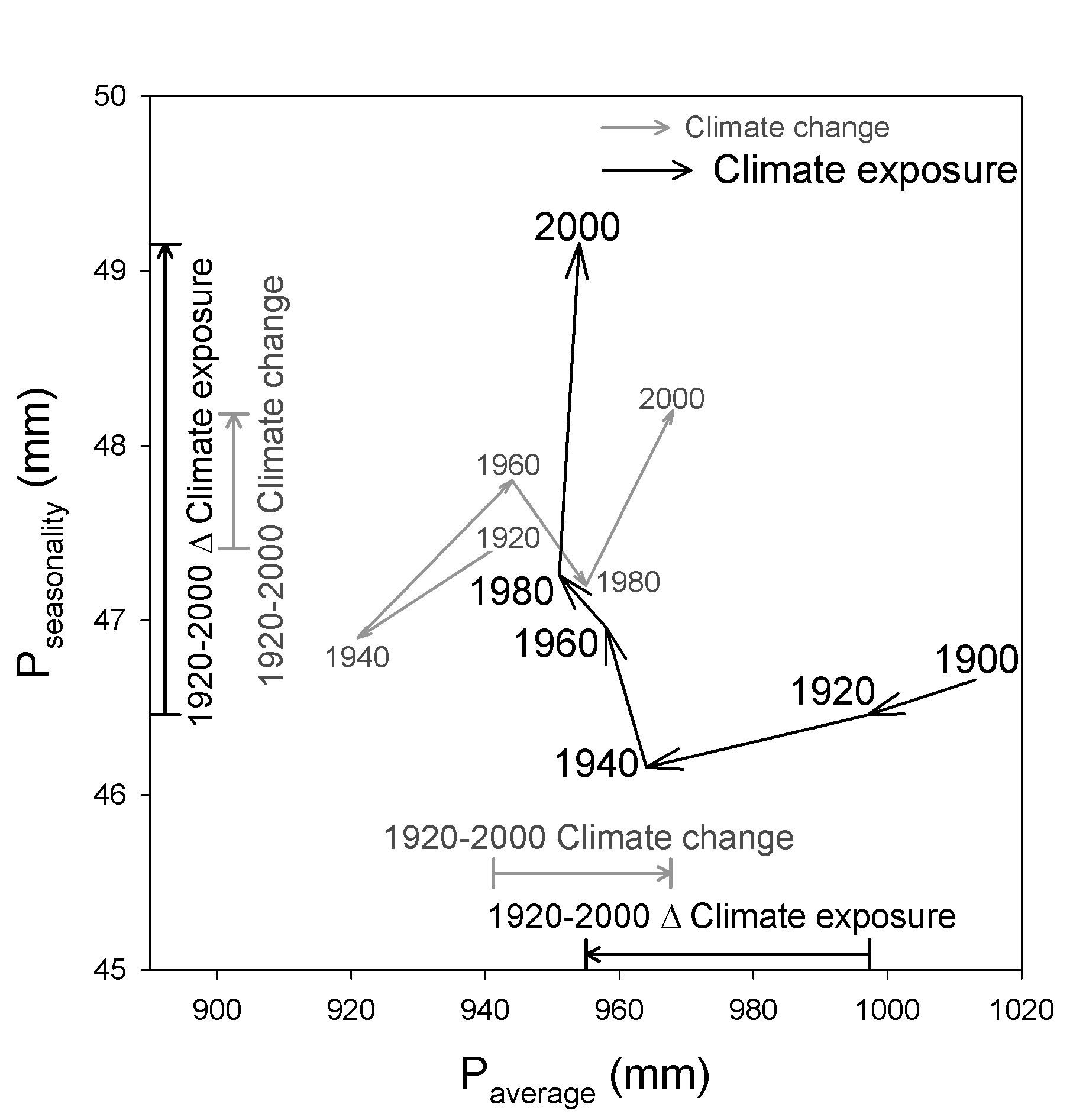


Figure S5. **Changes in climate conditions in the contiguous United States during the 20^th^ century averaged across 2728 counties (*Climate change*) and averaged across U.S. citizens (*Climate exposure*).** The x-axis represents total annual precipitation (mm) and the y-axis precipitation seasonality (mm). The arrows beside the axes represent the change in climate conditions between 1920 and 2000 for both time series. We estimated the climate conditions of each temporal horizon by averaging annual climate conditions of the preceding 20 years and, given the lack of climate data prior to 1900, we used the 1901-1920 climate averages in our analyses of both 1900 and 1920. The *Climate change* result for 1900 is therefore omitted while the *Climate exposure* results in 1900 and 1920 are based on the same climate conditions but different population sizes. See material and methods for details.
