# Supplementary material for: Demographic Amplification of Climate Change Experienced by the Contiguous United States Population during the 20th Century
Source: PLoS One. 2012 Oct 24;7(10):e45683. doi: 10.1371/journal.pone.0045683 (PMC3480346; doi:10.1371/journal.pone.0045683)
Supplement: Figure S6 — Spatial patterns of four climate variables for 2728 U.S. counties in five 20-year intervals during the 20th century. Counties shown in white were not included in the analyses because they did not have consistent census data or changed their geographical boundaries in the 20th century. In order to directly compare the spatial patterns between variables, each panel represents county z-scores based on the average and standard deviation of that variable throughout the century. A z-score of 0 represent the mean, whereas a value of 1 represent one standard deviation above the mean. (DOCX) [file pone.0045683.s006.docx]

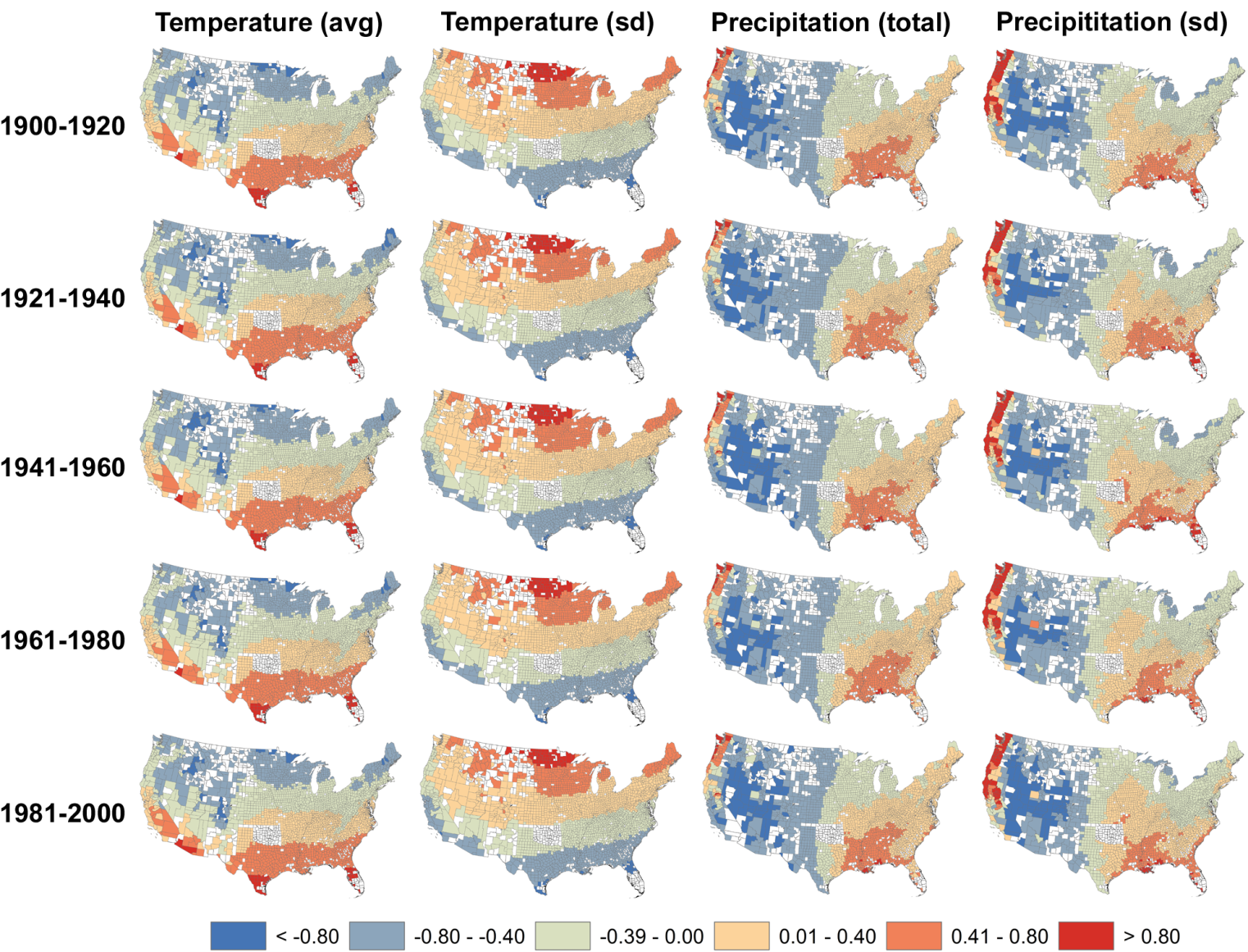


Figure S6. **Spatial patterns of four climate variables for 2728 U.S. counties in five 20-year intervals during the 20^th^ century.** Counties shown in white were not included in the analyses because they did not have consistent census data or changed their geographical boundaries in the 20^th^ century. In order to directly compare the spatial patterns between variables, each panel represents county z-scores based on the average and standard deviation of that variable throughout the century. A z-score of 0 represent the mean, whereas a value of 1 represent one standard deviation above the mean.
