# Supplementary material for: Demographic Amplification of Climate Change Experienced by the Contiguous United States Population during the 20th Century
Source: PLoS One. 2012 Oct 24;7(10):e45683. doi: 10.1371/journal.pone.0045683 (PMC3480346; doi:10.1371/journal.pone.0045683)
Supplement: Figure S7 — Standardized regression coefficients (Stdβ) of the four least important predictors of demographic growth rates for 2728 U.S. counties in five 20-year intervals during the 20th century (see Fig. 6 ). Counties shown in white were not included in the analyses because they did not have consistent census data or changed their geographical boundaries during the 20th century. See fig. 6 for the Stdβ of the four other variables used in this analysis. (DOCX) [file pone.0045683.s007.docx]

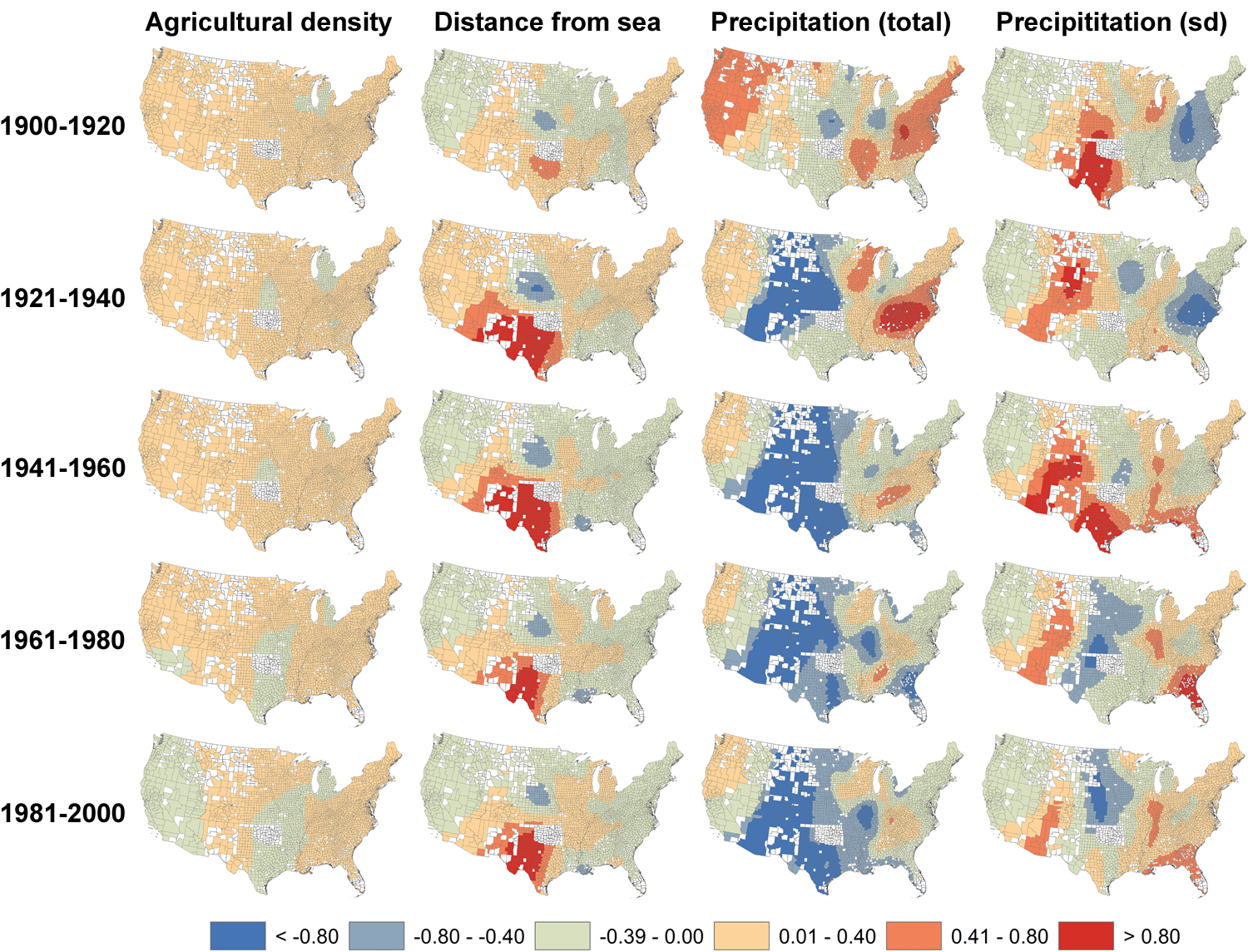


Figure S7. **Standardized regression coefficients (Stdβ) of the four least important predictors of demographic growth rates for 2728 U.S. counties in five 20-year intervals during the 20^th^ century (see Fig. 6).** Counties shown in white were not included in the analyses because they did not have consistent census data or changed their geographical boundaries during the 20^th^ century. See fig. 6 for the Stdβ of the four other variables used in this analysis.
