# Supplementary material for: Demographic Amplification of Climate Change Experienced by the Contiguous United States Population during the 20th Century
Source: PLoS One. 2012 Oct 24;7(10):e45683. doi: 10.1371/journal.pone.0045683 (PMC3480346; doi:10.1371/journal.pone.0045683)
Supplement: Table S1 — Model selection with Akaike information criterion (AIC) of stationary (OLS) and non-stationary (GWR) regression models predicting demographic growth rates for 2728 U.S. counties in the 20th century based on climatic (C) and/or non-climatic (NC) predictors. The AICc weigths of the GWRC+NC models were always one across all temporal horizons. The non-stationary nature of GWR was taken into account by adjusting the number of effective parameters (ranges of effective parameters across temporal horizons: GWRC+NC = 63.3–65.2; GWRC = 33.1–33.8; GWRNC = 22.9–39.8). (DOCX) [file pone.0045683.s008.docx]

1. **Evaluation of spatial non-stationarity**

We assessed the spatial stationarity of our demographic change models by comparing the predictive power of GWR models (non-stationary) with ordinary least square (OLS) regression models (stationary). In order to detect potential spurious correlations between climatic and non-climatic variables, we contrasted the spatial patterns of variable influences between models with climatic variables (GWR_C_, OLS_C_), non-climatic variables (GWR_NC_, OLS_NC_), or both (GWR_C+NC_, OLS_C+NC_). We used Akaike Information Criterion adjusted for small sample size (AICc) [[1](#_ENREF_1)]to evaluate the fit of each model (Table S1). The figures in the main text are all based on the GWR_C+NC_ models and the results from the GWR_C_ and the GWR_NC_ models can be found in Fig. S2 and Fig. S3.

Table S1. **Model selection with Akaike information criterion (AIC) of stationary (OLS) and non-stationary (GWR) regression models predicting demographic growth rates for 2728 U.S. counties in the 20^th^ century based on climatic (C) and/or non-climatic (NC) predictors.** The AICc weigths of the GWR_C+NC_ models were always one across all temporal horizons. The non-stationary nature of GWR was taken into account by adjusting the number of effective parameters (ranges of effective parameters across temporal horizons: GWR_C+NC_ = 63.3-65.2; GWR_C_ = 33.1-33.8; GWR_NC_ = 22.9-39.8).

| Predictive model | | 1901-1920 | | | 1921-1940 | | | 1941-1960 | | | 1961-1980 | | | 1981-2000 | | | |
| --- | --- | --- | --- | --- | --- | --- | --- | --- | --- | --- | --- | --- | --- | --- | --- | --- | --- |
|  | Adj-R^2^ | | AICc | ΔAICc | Adj-R^2^ | AICc | ΔAICc | Adj-R^2^ | AICc | ΔAICc | Adj-R^2^ | AICc | ΔAICc | | Adj-R2 | AICc | ΔAICc |
| GWR_C+NC_ | 0.57 | | -14067 | 0 | 0.49 | -15767 | 0 | 0.55 | -17038 | 0 | 0.61 | -18581 | 0 | | 0.66 | -19629 | 0 |
| GWR_C_ | 0.38 | | -13081 | 986 | 0.39 | -15316 | 451 | 0.38 | -16199 | 838 | 0.37 | -17271 | 1309 | | 0.37 | -17992 | 1636 |
| GWR_NC_ | 0.49 | | -13674 | 393 | 0.34 | -15148 | 619 | 0.33 | -16003 | 1035 | 0.48 | -17724 | 856 | | 0.54 | -18829 | 799 |
| OLS_C+NC_ | 0.37 | | -13075 | 992 | 0.21 | -14661 | 1106 | 0.27 | -15801 | 1237 | 0.39 | -17407 | 1173 | | 0.45 | -18403 | 1225 |
| OLS_C_ | 0.17 | | -12346 | 1720 | 0.19 | -14580 | 1188 | 0.15 | -15420 | 1617 | 0.18 | -16618 | 1963 | | 0.20 | -17393 | 2236 |
| OLS_NC_ | 0.35 | | -13020 | 1047 | 0.12 | -14373 | 1394 | 0.06 | -15132 | 1906 | 0.07 | -16316 | 2265 | | 0.13 | -17180 | 2448 |

Literature cited

1. Anderson DR, Link WA, Johnson DH, Burnham KP (2001) Suggestions for presenting the results of data analyses. Journal of Wildlife Management 65: 373-378.
